# Velpeau’s Elements of Operative Surgery

**Published:** 1846-05-01

**Authors:** 


*New Elements of Operative Surgery, by Alf. A. L. M. Velpeau, Prof. of Surgical Clinique, &c. Carefully revised, entirely remodelled, and augmented, with a treatise on minor Surgery; illustrated by over* 300 *engravings; accompanied with an atlas in quarto of twenty-two plates. First American from the last Paris edition. Translated by P. S. Townsend, M. D. &c. Augmented by several hundred pages of new matter, under the supervision of, and with notes and additions by Valentine Mott, M. D., etc.*

The second volume of this truly great work has lately been issued from the press of J. and H. G. Langley, New-York. Another volume is yet to appear, and is announced for October next. When completed it will constitute *the* great Surgical work of the age, embracing, in addition to the established principles of modern Surgical Science, the individual experience of two of the most eminent of living operative Surgeons. The author, Velpeau, who by the force of his talents and indomitable perseverance, despite every obstacle, has elevated himself to his present position, is, confessedly, at the head of French Surgery. Of the reputation of the senior American Editor as an operative Surgeon, it is supererogatory to speak. In short the names attached to the work as author, translator, and editor, afford an abundant guaranty of its value. That a critical analysis will reveal imperfections is to be expected : but we may say of books as of individuals, what one is perfect! We do not profess to have given either of the volumes which have been issued sufficient examination to attempt to point out particular excellencies or defects. An analytical review of all the volumes may be expected in a future number of our journal, by a writer fully competent to the task. We cannot refrain from expressing our regret that the translator, in announcing his motives for undertaking the work, should have allowed himself to be so far carried away by his enthusiastic veneration for the senior editor, as to indulge such excessive laudation of his genius and glory! It is to say the least a rank offense against good taste. Dr. Mott has obtained a reputation in operative surgery, of which the profession of this country have reason to feel proud, and which we presume, no one is disposed to depreciate. But that "no name that adorns the annals (of our country) either in the battle field, or in the councils of government, or in its diplomacy, has added more sterling reputation and abiding lustre to the intrinsic glory and future fame of America," (in our humble opinion) had better have been left, for the present, unsaid, more especially by one who takes occasion to call himself a kinsman. The apologetic statement that the eminent Professor has a "repugnance to self-laudation" is at variance with the impressions which they who listen to his instructions, bear away with them; and since the work passes under his supervision, we must, at all events, infer that he has no great repugnance to receiving laudation from others. These blemishes, however, have no reference to the practical value of the work, which we do not doubt is fully equal to what may reasonably be expected. The practitioner who wishes to keep pace with the progress of surgical knowledge, will of course purchase it. The price for the three volumes is $10,00. The last vol. will be accompanied by a quarto atlas of plates.

J. and H. G. Langley, N. Y. are the Publishers.

